# In Silico Study of the Mechanism of Binding of the N-Terminal Region of α Synuclein to Synaptic-Like Membranes

**DOI:** 10.3390/life10060098

**Published:** 2020-06-26

**Authors:** Carlos Navarro-Paya, Maximo Sanz-Hernandez, Alfonso De Simone

**Affiliations:** 1 Department of Life Sciences, Imperial College London, South Kensington, London SW7 2AX, UK; carlos.navarro-paya11@imperial.ac.uk (C.N.-P.); maximo.sanz10@imperial.ac.uk (M.S.-H.); 2 Department of Pharmacy, University of Naples “Federico II”, 80131 Naples, Italy

**Keywords:** α-Synuclein, membrane binding, disorder-to-order transition, coarse-grained simulations, Parkinson’s disease

## Abstract

The membrane binding by α-synuclein (αS), a presynaptic protein whose aggregation is strongly linked with Parkinson’s disease, influences its biological behavior under functional and pathological conditions. This interaction requires a conformational transition from a disordered-unbound to a partially helical membrane-bound state of the protein. In the present study, we used enhanced coarse-grained MD simulations to characterize the sequence and conformational determinants of the binding to synaptic-like vesicles by the N-terminal region of αS. This region is the membrane anchor and is of crucial importance for the properties of the physiological monomeric state of αS as well as for its aberrant aggregates. These results identify the key factors that play a role in the binding of αS with synaptic lipid bilayers in both membrane-tethered and membrane-locked conformational states.

## 1. Introduction

α-Synuclein (αS) is a 14 kDa protein that is highly abundant in the brain, where it localizes predominantly at the synaptic terminals [1,2,3]. The aggregation of αS is associated with the formation of Lewy Bodies in Parkinson’s disease (PD) and in other synucleinopathies [4,5,6,7,8]. Genetic links exist between αS and hereditary forms of early onset PD, including point mutations, duplications and triplications of the αS encoding gene [9,10,11]. Fibrillar aggregates of the non-amyloid-β component (NAC) region (residues 65–95) of αS are also found in the context of Alzheimer’s disease (AD), multiple system atrophy [12], dementia with Lewy bodies [13], and other synucleinopathies [7].

The functional role of αS, however, remains unclear [14]. The predominant localization of αS at the presynaptic terminals has indicated that it may be involved in various neuronal processes. The main body of evidence indicates that it has a role in regulating the trafficking of synaptic vesicles (SVs) [15,16,17,18], including a chaperone role in SNARE formation via an interaction with VAMP-2 at the surface of SVs [15,16,18]. Moreover, because of its ability to promote vesicle–vesicle interactions, clustering and fusion, which have been observed both in vitro [19,20] and in vivo [18], αS has been associated with the maintenance of SV pools [21,22] and with ER-to-golgi vesicle trafficking [17,23]. Additional putative roles include the mitigation of oxidative stress in mitochondria [24] and the interaction and regulation of ATP synthetase at the surface of mitochondrial membranes [25]. Taken together, the majority of the suggested functions of αS involve the crucial step of binding to cellular membranes, a process that is tightly regulated in vivo [26], and that affects the rate of αS aggregation [27,28,29] and the toxicity of its aggregates [22,30].

In the cytoplasm, αS is disordered and monomeric [31] and shows negligible secondary structure content throughout its sequence [32,33]. However, upon acetylation, the N-terminal residues adopt an increased α-helix character [32]. Upon binding with biological membranes, αS becomes highly enriched in α-helical structure [19,34,35], a transition that is promoted by a series of seven imperfect repeats located in the region 1–90 of the sequence that encode for lipophilic class A2 amphipathic α-helical segments [35]. These segments enable αS to adapt its binding properties to a large variety of amphipathic surfaces, ranging from detergent micelles to lipid bilayers as well as to the water–air interface [36]. NMR studies of αS bound to micelles have revealed a broken α-helix conformation formed by two helical segments in an antiparallel topological arrangement (residues 3–37 and 45–92) [34] whilst studies of the membrane-bound state reported mainly a single helix formed throughout the region 1–97 [37]. However, an emergent view suggests that a multitude of possible conformations are adopted by αS at the surface of membranes [19,34,38], including oligomeric states that promote self-assembly and aggregation [15,39].

Upon interaction with synaptic-like vesicles, three different regions of αS were found to exhibit distinctive structural and dynamical properties [40]. These regions include the N-terminal segment (residues 1–25), which is the primary membrane-anchoring region that adopts the conformation of a stable α-helix tightly bound to the surface of acidic lipid bilayers. A second region of the protein, spanning residues 26 to 97, was found to have an intermediate binding affinity for synaptic membranes and plays the role of “sensor” of the lipid properties that influences the overall membrane affinity of αS. Finally, the negatively charged C-terminal region (residues 98–140) was shown to remain mostly unbound from the membrane surface [40] and induces conformational changes in the protein upon calcium binding, thereby promoting strong co-localization with synaptic vesicles in synaptosomes [41].

The present study exploits an in silico research framework to study the details of the complex membrane binding process by αS, using enhanced molecular dynamics (MD) simulations based on coarse-grained force fields [42]. To this end, we adapted the Martini 3 force field to address specific questions about the binding modes of the 30 N-terminal residues of αS with synaptic membranes. We showed that this region has specific sequence properties that enable membrane binding in both helical conformation and in extended-tethered form. Deletion constructs known to alter the binding affinity of αS to membranes were indeed shown to affect the interaction of both conformational states in a correlated manner. The simulations also showed evidence for the role played by Phe 4 as a key promoter of the membrane interaction by αS. Overall, our study characterizes the nature of the balance between sequence and conformational properties in driving the interaction of this region with the membrane surface.

## 2. Materials and Methods

### 2.1. Simulation Setup

Simulations of the binding of the N-terminal region of αS to synaptic-like membranes were run using the GROMACS 4.6.7 platform [43] and a modified version of the Martini 3 force field [44]. In order to study the interaction of αS with synaptic-like membranes employed in previous experimental studies [40], we simulated a membrane composed of DOPE, DOPS and DOPC lipids in a 5:3:2 (w/w) ratio (167 lipid molecules in total) and the acetylated N-terminal region of αS (residues 1–30 designated as αS_1–30_, Table 1). The membrane was generated using the python script insane.py [45] and solvated with Martini water models and Cl^−^ and Na^+^ ions to a salt concentration of 150 mM. Backbone termini at both ends of the protein were modeled as uncharged, including the acetylated first residue (M1) that was neutralized with the -nt flag and last residue (Ala 30) of αS_1–30_, which was also kept neutral. In addition to simulations of αS_1–30_, we run control simulations using the C-terminal region of the protein (αS_111–140_), which was modeled with an uncharged first residue (Gly 111) and a charged C-terminal residue (Ala 140). The conformations of αS_1–30_ or αS_111–140_ were generated using the martinize.py script starting from full atomic models of αS in helical or extended-disordered states. In each simulation, the peptide’s center of mass in the starting conformation was positioned at a distance of 4 nm from the lipid bilayer. The system was then equilibrated at different temperatures, using a series of 10 ns MD simulations in the NPT ensemble with an integration time step of 10 fs. Thermal equilibration was computed using the Velocity-rescale thermostat [46]. Fifteen equilibrations were run at the following temperatures: 310 K, 320 K, 330 K, 340 K, 350 K, 360 K, 370 K, 380 K, 390 K, 400 K, 410 K, 420 K, 430 K, 440 K, 450 K, with a coupling constant of 2 ps and by separately coupling water molecules, Na^+^ and Cl^−^ ions, protein and the membrane. Pressure was equilibrated at 1 bar using a semi-isotropic Berendsen barostat [47], coupling the xy and z axis separately with a relaxation time of 12 ps and compressibility of 3 × 10^−4^ bar^−1^.

Subsequently, sampling simulations were run using a timestep of 20 fs, for 15 independent samplings based on the same temperature range of the equilibration step (310 K to 450 K), and using the Velocity-rescale thermostat [46] and the Parrinello-Rahman barostat [48]. Electrostatic interactions were treated using the reaction field method with a Coulomb cut-off of 1.1 nm, whereas a cut off of 1.1 nm was used for van der Waals interactions. Bond lengths and sidechain angles were constrained using the Lincs algorithm [49]. Each simulation in the sampling phase was 4.8 μs with a total simulation sampling of 1368 μs. Details of the various simulations run in this study are reported in Table 1. Convergence of the simulations was checked by dividing the trajectories in three consecutive parts and by comparing the calculated observables in these independent segments.

### 2.2. CG Implementation

We modified the standard Martini 3 CG force field to restrain the protein in two states, namely, α-helical or extended-disordered conformations. In the context of the interaction with the membrane surface, these conformations mimic the final bound state (α-helical) and the initial tethered state (extended-disordered) in which the protein is absorbed onto the membrane surface in a disordered manner. To restrict the conformational variability of αS to these conformations, we restrained the angle between three consecutive backbone particles i, j and k (θ_ijk_) in the Martini 3 force field using Gaussian function energy potential. The angle θ_ijk_ between three particles is defined as

(1) $$\theta_{\mathrm{ijk}}=\cos^{-1}\left(\frac{\vec{\mathrm{ij}}\cdot \vec{\mathrm{kj}}}{\left\|\vec{\mathrm{ij}}\|\|\vec{\mathrm{kj}}\right\|}\right)$$

and the restraining Gaussian potential:

(2) $$V_{\theta }=-K_{\mathrm{ijk}}e^{\frac{{\left(\theta_{\mathrm{ijk}}-{\;\theta }_{\min}\right)}^{2}}{\sigma }}$$

where $K_{\mathrm{ijk}}$ is the force constant of the Gaussian potential, $\theta_{\min}$ defines the minimum of the potential energy (adopting 96° and 124° in helical and extended-disordered conformations, respectively) and σ is a constant that determines the width of the Gaussian minimum. The value for σ was set to 14° for all simulations (both helical and extended-disordered conformations). Control simulations performed by using σ values of 114° generated consistent results (Figure S1). The angle restraining force in our potential is:

(3) $$\vec{F_{\theta }}=-\frac{{\mathrm{dV}}_{\theta }}{d\vec{r}}=-\frac{{\mathrm{dV}}_{\theta }}{d\theta }\frac{d\theta }{d\vec{r}}=\frac{2K_{\mathrm{ijk}}(\theta_{\mathrm{ijk}}-{\;\theta }_{\min})e^{\frac{{\left(\theta_{\mathrm{ijk}}-{\;\theta }_{\min}\right)}^{2}}{\sigma }}}{\sigma }\frac{d\theta }{d\vec{r}}$$

where $\vec{r}$ denotes the atomic coordinate vector of each particle.

Our modified version of Martini 3 also employed the restraint of dihedral angles between four consecutive backbone particles i, j, k and l (φ_ijkl_), ranging from −180 to 180 degrees in value, and is defined as the four-quadrant inverse tangent:

(4) $$\cos\left(\varphi_{\mathrm{ijkl}}\right)=\frac{\left(\vec{\mathrm{ij}}\times \vec{\mathrm{jk}}\right)\cdot \left(\vec{\mathrm{jk}}\times \vec{\mathrm{lk}}\right)}{\left\|\vec{\mathrm{ij}}\times \vec{\mathrm{jk}}\|\;\|\vec{\mathrm{jk}}\times \vec{\mathrm{lk}}\right\|}$$

(5) $$\sin\left(\varphi_{\mathrm{ijkl}}\right)=\frac{\vec{\mathrm{jk}}\cdot \left[\left(\vec{\mathrm{ij}}\times \vec{\mathrm{jk}}\right)\times \left(\vec{\mathrm{jk}}\times \vec{\mathrm{lk}}\right)\right]}{\left\|\vec{\mathrm{jk}}\|\;\|\vec{\mathrm{ij}}\times \vec{\mathrm{jk}}\|\;\|\vec{\mathrm{jk}}\times \vec{\mathrm{lk}}\right\|}$$

(6) $$\varphi_{\mathrm{ijkl}}=\;\mathrm{atan}2\left(\sin\left(\varphi_{\mathrm{ijkl}}\right),\cos\left(\varphi_{\mathrm{ijkl}}\right)\right)$$

The Gaussian potential is constructed as before:

(7) $$V_{\varphi }=-K_{\mathrm{ijkl}}e^{\frac{{\left(\varphi_{\mathrm{ijkl}}-{\;\varphi }_{\min}\right)}^{2}}{\sigma }}$$

where $\varphi_{\min}$ defines the minimum of the potential energy (set to 60° and 100° for helical and extended-disordered conformations, respectively). The dihedral potential is implemented with two additional values of $\varphi_{\min}$, specifically set at $\varphi_{\min}\;\pm \;360{}^{\circ}$, in order to avoid discontinuities at the periodic boundaries of the atan2 function. The value for σ was set to 14° for all simulations (both helical and extended-disordered conformations), and control simulations run with σ values of 114° resulted in consistent data (Figure S1). The dihedral restraining force in our potential is

(8) $$\vec{F_{\varphi }}=-\frac{{\mathrm{dV}}_{\varphi }}{d\vec{r}}=-\frac{{\mathrm{dV}}_{\varphi }}{d\varphi }\frac{d\varphi }{d\vec{r}}=\frac{2K_{\mathrm{ijkl}}(\varphi_{\mathrm{ijkl}}-{\;\varphi }_{\min})e^{\frac{{\left(\varphi_{\mathrm{ijkl}}-{\;\varphi }_{\min}\right)}^{2}}{\sigma }}}{\sigma }\frac{d\varphi }{d\vec{r}}$$

## 3. Results

### 3.1. Conformational Dependency in the Membrane Binding of N-Terminal Region αS

In order to sample the binding modes of the N-terminal region (residues 1–30) of αS (αS_1–30_) with DOPE:DOPS:DOPC lipid bilayers, we performed a series of CG MD simulations using a modified version of the Martini 3 force field (see Methods). In particular, the conformations of the backbone were restrained to adopt two main basins. The first was an extended-disordered conformation describing the initial “tethered” state of the protein absorbed onto the membrane surface in an amorphous manner. The second was a helical conformation describing the final “locked” membrane bound state, which for this region is a stable α-helix anchoring the protein onto the membrane surface [36].

**Figure 1:**
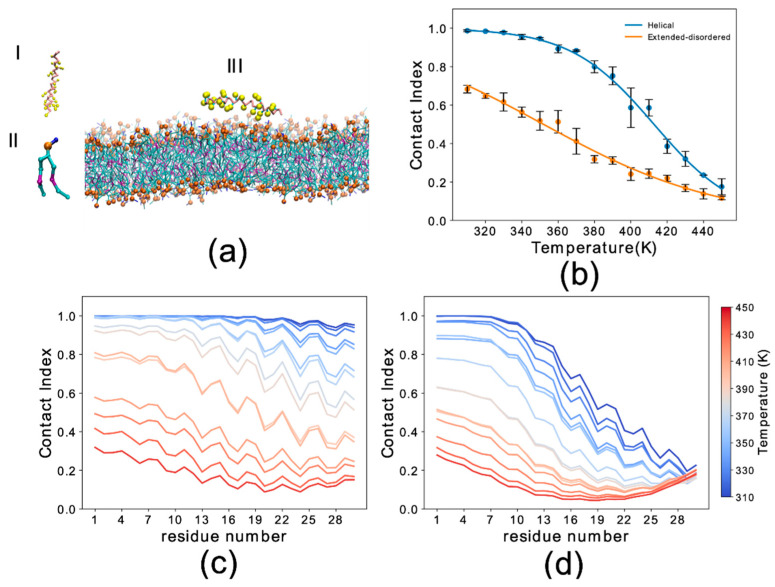
Simulation setup and contact index comparison between the helical and extended-disordered conformations. (**a**) Representative setup of the simulations. (I) αS_1–30_ molecule in the Martini 3 force field in helical conformation. Backbone and side chain particles are shown in pink and yellow, respectively. (II) Representative lipid molecule (DOPE). The phosphate group is shown in orange. (III) Lipid bilayer (DOPE, DOPS and DOPC in a 5:3:2 w/w ratio) and an αS_1–30_ molecule in helical conformation. (**b**) Membrane-binding melting curves showing the global contact index as a function of the temperature of the simulation. Blue and orange lines report the melting curves of αS_1–30_ binding to DOPE:DOPS:DOPC lipid bilayer, with the protein in helical and extended-disordered conformations, respectively. Error bars report the standard deviation between three segments of the simulation. (**c**,**d**) Residue specific contact indexes in temperatures ranging from 310 K (dark blue) to 450 K (dark red), with a step increment of 10 K. Contact indexes for αS_1–30_ binding to DOPE:DOPS:DOPC lipid bilayer in helical (**c**) and extended-disordered (**d**) conformations.

αS_1–30_ was modeled in the acetylated form of the protein and with an uncharged C-terminal residue 30. In addition to the protein and the DOPE:DOPS:DOPC lipid bilayer (167 lipid molecules), the systems included 8686 to 8981 water molecules (Table 1) and Na^+^ and Cl^−^ ions at a concentration of 150 mM. In all the simulations, the starting configuration of the protein was positioned at 4 nm from the membrane (Figure 1a). Systems were equilibrated in the NPT ensemble for 10 ns until convergence of the area occupied by the lipids. Samplings of each system, duplicated to sample both the helical and the extended-disordered states, were carried out at 15 different temperatures (collectively ranging from 310 K to 450 K with a 10 K step increase). Each sampling was run for 4.8 μs, which collectively amounted to a total sampling of 1368 μs in this study.

We first analyzed the trajectories to obtain the probability of membrane interaction by each residue of the protein using the membrane contact index, which is calculated from the minimum distance between Cα atoms of the protein residues and phosphate atoms of the membrane. Membrane contacts were attributed based on a threshold of 1 nm. Residue-specific contact indexes were calculated for each frame of the sampling and averaged across the whole trajectory. The average of the residue contact indexes along the sequence provided the global contact index, which was plotted as a function of temperature to plot melting curves of the interaction with the membrane (Figure 1b). In addition to providing information on the membrane binding affinity, melting curves were also used to estimate the convergence of the simulations by comparing the data from three independent trajectory segments (Figure S2).

The results indicated that the backbone conformation has a significant influence on the binding properties of αS_1–30_. In particular the helical conformation strongly favors the membrane-binding of this region of αS, resulting in an increase of 60 K in the melting temperature when compared to the extended-disordered conformation (Figure 1b). At the lowest temperatures the global contact index reached a value of 1 for the helical state, indicating complete binding of αS_1–30_ at 310 K. In contrast, the maximum global contact index for the extended-disordered conformation was only 0.69. Another difference in the binding modes of the two conformations is that membrane contacts at 310 K were uniform throughout the sequence for the helical conformation (Figure 1c), with contact indexes showing a sequence periodicity recalling the nature of the α-helix structure. By contrast, in the extended-disordered conformation, strong membrane contacts were only observed in the initial seven residues (Figure 1d), with the rest of the sequence showing a progressive decrease in binding. As the temperature increases from 310 K (dark blue) to 450 K (dark red), the probability of contacts is reduced non-uniformly across the sequence in both types of conformations, with the initial 11 and 7 residues establishing the strongest contacts overall in the case of helical and extended disordered conformations, respectively.

### 3.2. Sequence Dependencies in the Membrane Affinity of αS_1–30_

**Figure 2:**
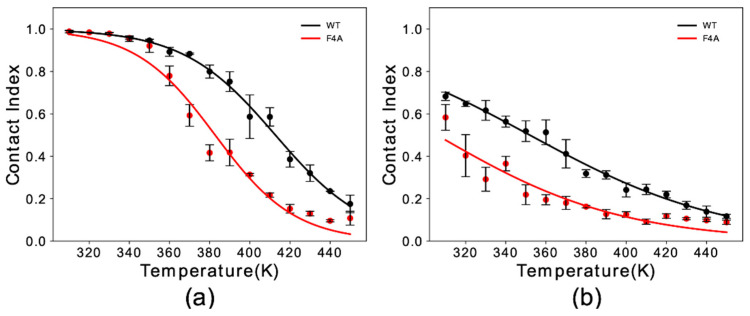
Membrane binding melting curves of binding to DOPE:DOPS:DOPC lipid bilayers by WT and F4A αS_1–30_. The curves illustrate the dependency of the global contact index as a function of the temperature of the simulation. Data for WT and F4A αS_1–30_ are shown in black and red, respectively. Panels (**a**,**b**) report data for αS_1–30_ in helical and extended conformation, respectively. Error bars report the standard deviation between three segments of the simulation.

Experimental studies indicated a key role of residue Phe 4 in the membrane binding properties of αS [31]. Here, we simulated F4A αS_1–30_ to study the effect of this mutation with our in silico membrane-binding assay. Our results showed that F4A induces a dramatic reduction in the membrane affinity of αS_1–30_ (Figure 2). The impairment of the membrane binding by this mutation was observed for both helical (Figure 2a) and extended-disordered states (Figure 2b), with a reduction in the melting temperature of 31 K and 42 K, respectively. The data therefore indicate that F4 has a role in both stabilizing the final helical bound state of the protein at the surface of DOPE:DOPS:DOPC lipid bilayers as well as the initial tethering process.

Structural studies by solid-state NMR [50] and in vitro binding assays [51] have evidenced the crucial role of the first 11 residues of the αS sequence in stabilizing the membrane binding of the protein. Constructs of αS with progressive truncations of the N-terminal residues were found to have altered membrane-binding affinities, with the deletion of residues 2–11 generating the strongest impairment in the binding [51]. We assessed in silico the membrane interaction of truncated versions of αS_1–30_, both in the helical and extended-disordered states.

**Figure 3:**
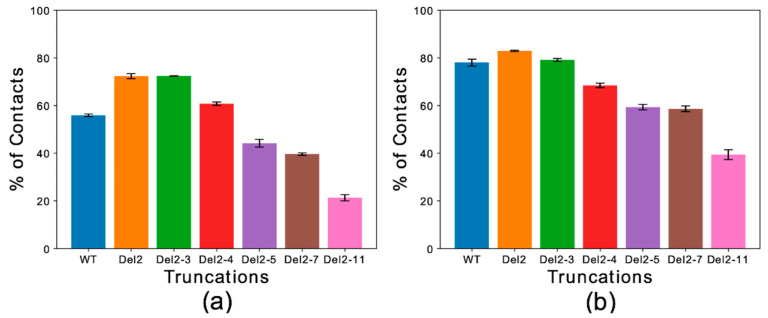
Temperature-averaged global contact index (TAGCI). (**a**,**b**) The membrane interactions of different deletion constructs αS_1–30_ were simulated to mimic the conditions of previous experimental studies [51]. Helical (**a**) and extended-disordered (**b**) conformations were simulated across 15 temperatures. The TAGCI reports the average contact indexes by the first 15 residues of each construct and further averaged across the 15 temperatures employed in this study. The construct nomenclature reports the residues that have been deleted from the WT sequence.

**Figure 4:**
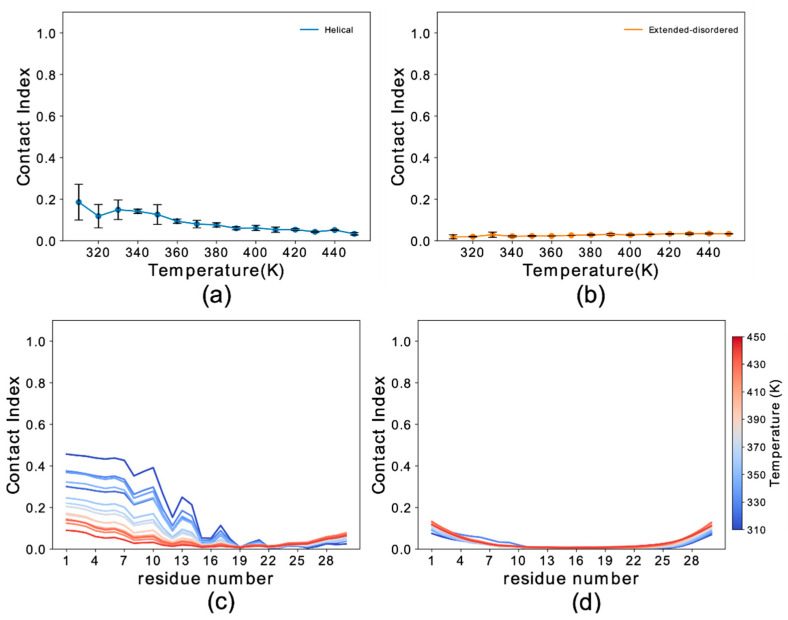
Binding properties of αS_111–140_ to DOPE:DOPS:DOPC lipid bilayers. (**a**,**b**) Membrane-binding melting curves of αS_111–140_ calculated from plotting the global contact index as a function of the temperature of the simulation. Helical and extended-disordered conformations are shown in panels (a) and (b), respectively. Error bars report the standard deviation between three segments of the simulations. (**c**,**d**) Residue specific contact indexes in the range of temperatures going from 310 K (dark blue) to 450 K (dark red) at step increment of 10 K. Plots for αS_111–140_ binding to DOPE:DOPS:DOPC lipid bilayer in helical (**c**) and extended-disordered (**d**) conformations are shown.

Global contact indexes were calculated by using a window of 15 residues starting at the first amino acid of each construct. The resulting melting curves (Figure S3) were further condensed into a temperature-averaged global contact index (TAGCI) to provide a direct comparison between the membrane affinities of different αS constructs using a single value. TAGCI values of helical (Figure 3a) and extended conformations (Figure 3b) of the αS constructs featuring different levels of N-terminal truncation were in excellent agreement with experimental data [51]. The results indicate that the helical conformation maintains a higher membrane-binding affinity across all the constructs with respect to the extended-disordered state. Some sequence-specific patterns in the binding affinities of the truncated constructs were also observed. In particular, the deletion of residues Asp 2 and Val 3 from the sequence (Del-2 and Del-2-3) induced stronger membrane binding, as also observed experimentally [51]. This observation is likely associated with the removal of a negatively charged residue from the sequence. This effect was more prominent for the extended-disordered conformations, suggesting a negative regulation by Asp 2 of the tethering of αS to acidic membranes. Larger truncations generated a decrease in the binding affinity of αS in both helical and extended-disordered conformations, with the strongest effects found in the construct Del-2-11, which is in line with the experimental data [51]. Taken together, the data in the literature [50,51] and the present simulations highlight the key role of the first 11 residues of αS in the membrane binding of αS. In addition, the correlated patterns in helical (Figure 3a) and extended-disordered (Figure 3b) conformations indicate that the effects of the truncations are conformationally independent, thereby highlighting the intrinsic role of the sequence properties of αS_1–30_ in the membrane binding. The intrinsic role of the N-terminal sequence is further corroborated by control simulations of the region spanning the last 30 residues of αS (αS_111–140_), indicating a significant difference in the binding properties of αS_1–30_ and αS_111–140_. In particular, αS_111–140_ showed essentially no membrane affinity and a negligible conformational effect when comparing helical versus extended-disordered conformations (Figure 4).

## 4. Discussion

The interaction of αS with cellular membranes is central to defining the properties of its biological form [52]. Binding to synaptic membranes is a common step in the majority of the putative functions of αS [36,53] as well as a fundamental property of αS aggregates forming under pathological conditions. It has been shown that membrane binding influences the kinetics of αS aggregation, with both acceleration and inhibition being observed under different experimental conditions [28,54]. Membrane binding also modulates the properties of αS oligomers, whose toxicity is promoted by the binding and disruption of synaptic membranes [55]. Under physiological conditions, αS establishes a crucial interaction with SVs [22,36] that has been implicated in a number of its putative functions [15,16,18,20,22]. Several experimental studies have demonstrated that the N-terminal anchor of αS is the primary binding region for the interaction with the membrane component of SVs [19,40,51]. Here, we investigated this process by means of a simulation platform that enables analysis of the sequence and conformational dependencies of the binding of αS_1–30_ at a residue-specific resolution, with lipid bilayers mimicking the membrane component of SVs. Our results show that the interaction of αS_1–30_ with DOPE:DOPS:DOPC lipid bilayers is largely dependent on the conformation adopted by the protein, with the helical state having stronger binding affinity than the extended-disordered conformation. Our data also provide evidence that the sequence properties of αS_1–30_ crucially influence its binding modes to synaptic membranes. Indeed, simulation of the C-terminal sequence, αS_111–140_, resulted in negligible membrane interactions for both helical and extended-disordered conformations. Moreover, the simulations of αS_1–30_ constructs with progressive N-terminal truncations revealed trends in membrane affinities that are independent from the protein conformations. This result indicates that while the helical conformation generally promotes stronger membrane interactions, the sequence of αS_1–30_ is the key driver for the overall membrane binding by αS_1–30_. A key residue of this sequence is Phe 4, which was shown to anchor αS_1–30_ to the membrane in both types of conformations, suggesting that this residue is fundamental for both membrane-tethering and membrane-locking steps.

In conclusion, taken together, our data reveal the importance of the sequence properties of the N-terminal region of αS in promoting strong and adaptable binding to synaptic membranes. This region of αS has specificity to tether on the surface of acidic membranes in a disordered extended conformation, while the amphipathic α-helical conformation promotes the locking to the membrane surface. The mechanism of membrane interaction of this region is fundamental for the biological behavior of αS because it induces strong binding affinity without requiring a transmembrane helix. This topological property promotes a degree of reversibility in the membrane interaction by αS, a crucial characteristic that enables αS to bind with multiple membranes at the synaptic termini. Our data suggest that such binding reversibility is at least in part associated with the ability of αS_1–30_ to interact with lipid bilayers in both helical and extended-disordered conformations. However, this binding versatility, which we postulate has functional relevance, can also be utilized by αS oligomers in aberrant processes; indeed, one of the key steps for the toxicity of these aggregates is the membrane anchoring by the N-terminal region [55]. The adaptability of binding by the N-terminal region in both membrane-locked and membrane-tethered states can therefore be exploited by oligomeric species to promote binding promiscuity to different synaptic membranes and organelles, generating downstream effects leading to cellular toxicity.

## Figures and Tables

**Table 1:** Simulation details.

Name	Protein Sequence	Nr of Waters
WT αS_1–30_	MDVFMKGLSKAKEGVVAAAEKTKQGVAEAA	8954
Del 2 αS_1–30_	MVFMKGLSKAKEGVVAAAEKTKQGVAEAA	8949
Del 2–3 αS_1–30_	MFMKGLSKAKEGVVAAAEKTKQGVAEAA	8947
Del 2–4 αS_1–30_	MMKGLSKAKEGVVAAAEKTKQGVAEAA	8957
Del 2–5 αS_1–30_	MKGLSKAKEGVVAAAEKTKQGVAEAA	8964
Del 2–7 αS_1–30_	MLSKAKEGVVAAAEKTKQGVAEAA	8963
Del 2–11 αS_1–30_	MKEGVVAAAEKTKQGVAEAA	8981
CT αS_111–140_	GILEDMPVDPDNEAYEMPSEEGYQDYEPEA	8916
F4A αS_1–30_	MDVAMKGLSKAKEGVVAAAEKTKQGVAEAA	8696
σ 114° αS_1–30_	MDVFMKGLSKAKEGVVAAAEKTKQGVAEAA	8686

## Supplementary Materials

The following are available online at https://www.mdpi.com/2075-1729/10/6/98/s1, Figure S1: Effects of the variation in the σ of the restraining potential, Figure S2: Simulations convergence, Figure S3: Membrane interactions by different deletion constructs αS_1–30_.
