# Supplementary material for: In Silico Study of the Mechanism of Binding of the N-Terminal Region of α Synuclein to Synaptic-Like Membranes
Source: Life (Basel). 2020 Jun 26;10(6):98. doi: 10.3390/life10060098 (PMC7344899; doi:10.3390/life10060098)
Supplement: Supplementary file 1 [file life-10-00098-s001.pdf]

life

MDPI

Supplementary Material

In silico study of the mechanism of binding of the N-terminal region of α Synuclein to synaptic-like membranes

Carlos Navarro-Paya[1], Maximo Sanz-Hernandez[1], Alfonso De Simone[1,2,*]

[1] Department of Life Sciences, Imperial College London, South Kensington, SW7 2AX UK;

[2] Department of Pharmacy, University of Naples “Federico II”, via D. Montesano 49 Naples, 80131 Italy;

* Correspondence: adesimon@imperial.ac.uk

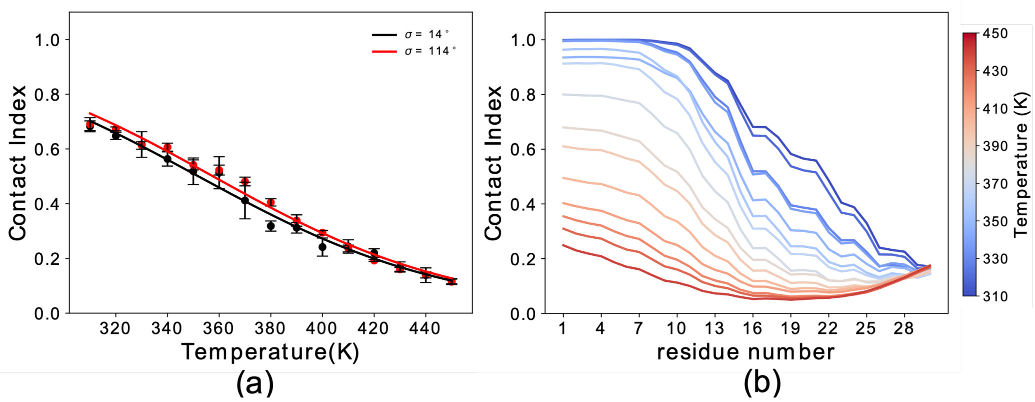

Figure S1. Effects of the variation in the σ of the restraining potential. We assessed if the conformational properties of the extended-disordered conformation are influenced by different values of σ employed in both angle and dihedral restraints. A) Membrane-binding melting curves calculated from plotting the global contact index as a function of the temperature of the simulation. The curves calculated from simulations using σ values of 14° (black) and 114° (red) are largely similar. B) Residue specific contact indexes in the range of temperatures going from 310K (dark blue) to 450K (dark red) at step increment of 10K. Plots for αS_{1-30} binding to DOPE:DOPS:DOPC lipid bilayer in the extended-disordered conformations are shown. These simulations were run with a σ value of 114°. Data using a σ value of 14° are shown in Fig. 1D.

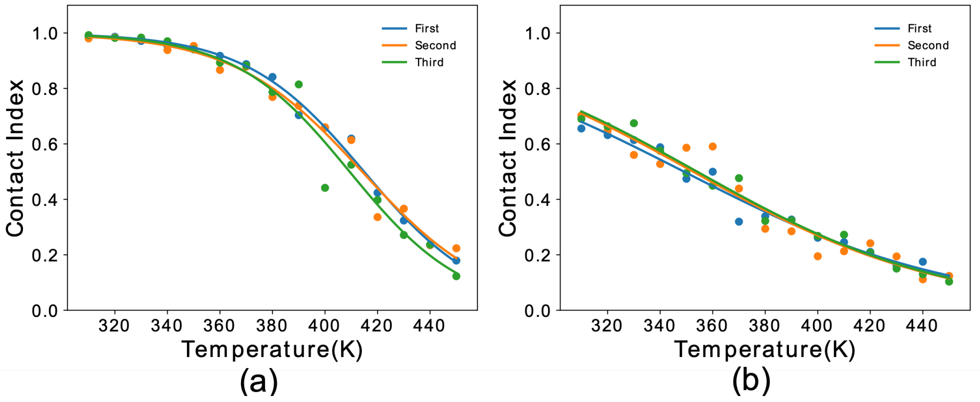

Figure S2. Simulations convergence. The convergence of the simulations has been assessed by dividing the trajectories in three equivalent and consecutive segments and by comparing observables calculated in these samplings. **(a-b)** Membrane-binding melting curves calculated from plotting the global contact index as a function of the temperature of the simulation. Plots for $\alpha S_{1\text{-}30}$ binding to DOPE:DOPS:DOPC lipid bilayer in the helical **(a)** and extended-disordered (**b)** conformations are shown. First, second and third segments of the simulations are shown in orange blue and green, respectively.

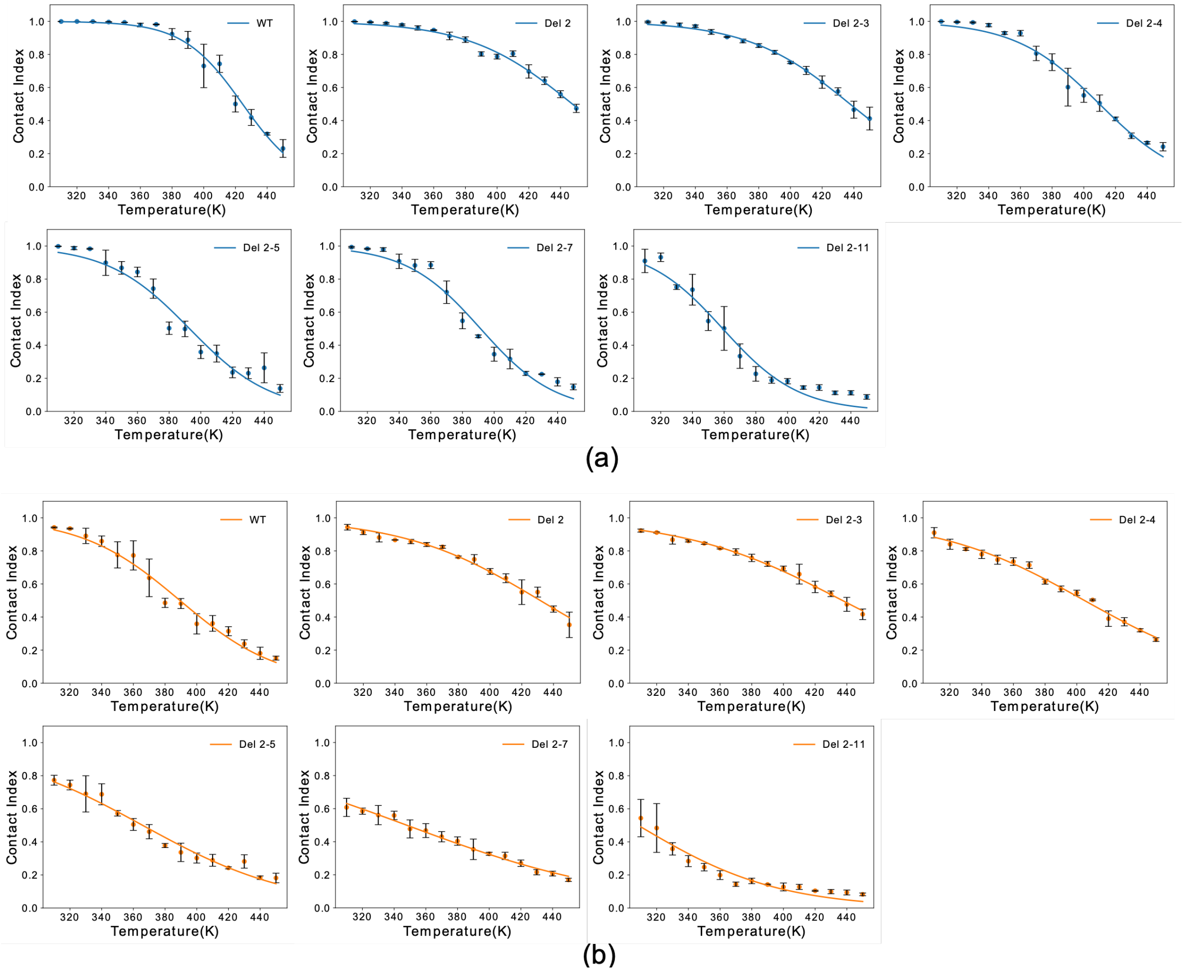

Figure S3. Membrane interactions by different deletion constructs αS_{1-30}. Membrane-binding melting curves calculated from plotting the global contact index as a function of the temperature of the simulation. Plots for the binding to DOPE:DOPS:DOPC by various αS constructs in the helical **(a)** and extended-disordered **(b)** conformations are shown. These plots are calculated by considering only the first 15 residues of each construct.

© 2020 by the authors. Submitted for possible open access publication under the terms and conditions of the Creative Commons Attribution (CC BY) license (http://creativecommons.org/licenses/by/4.0/).
